# Accelerated Use of Non-Surgical Techniques for Nevi Removal: Primum Non-Nocere

**DOI:** 10.5826/dpc.1104a117

**Published:** 2021-10-01

**Authors:** Seçil Vural, Bengü Nisa Akay, Arda Yaycıoğlu, Seher Bostancı

**Affiliations:** 1 Koç University, School of Medicine, Department of Dermatology and Venereology; 2 Ankara University, Faculty of Medicine, Department of Dermatology and Venereology

**Keywords:** melanoma, nevus, skin neoplasms, cosmetic dermatology

## Introduction

The desire to look more attractive has always been an undeniable demand in every society. With the effect of social media and the continuous use of filtered selfies, the longing for perfection has reached its climax.

Nowadays, increasingly more patients apply to dermatology clinics to eliminate skin imperfections, including nevi. We recommend removal of skin lesions performed in the classical and safe approach, and to perform histopathological examination on removed tissue. Conventional elliptic surgical removal may however heal with linear scarring, which is sometimes perceived as unsatisfactory by patients. In recent years, alternative methods such as ablative lasers have been introduced as a treatment option to destroy nevus cells near the skin surface to optimize cosmetic outcome with reduced scarring risk. Although laser removal is a feasible and charming option, the technique’s crucial pitfalls should not be disregarded. The main risk in destroying a nevus with ablative methods is the possibility of removing a melanoma. While early recognition and complete excision of melanoma is curative, advanced stages are associated with a high mortality rate, despite the progress in treatment modalities. Misinterpretation of melanoma for a nevus may lead to delayed diagnosis of an advanced/metastatic melanoma. Another potential risk of nevi laser removal is the malignant transformation of the remaining nevus cells into a melanoma. Unfortunately, these theoretical scenarios exist more often than we may think and have tragic consequences.

### Case 1

**Figure 1:**
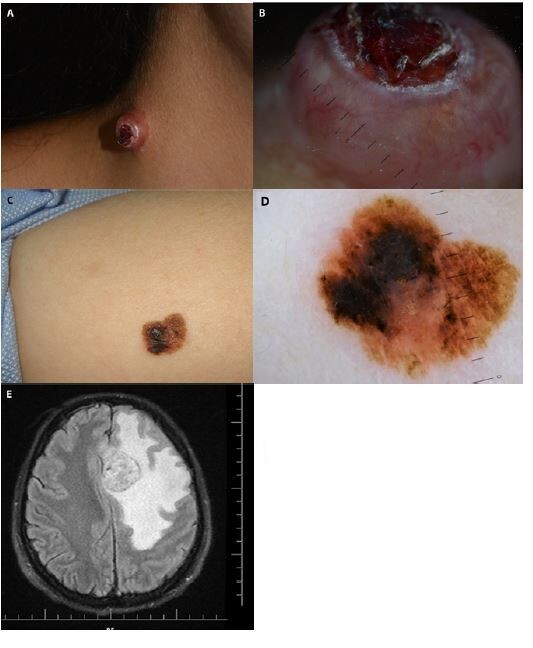
Case reports. (A) Case 1: 18-year-old woman diagnosed with pathology confirmed nodular melanoma with a Breslow thickness of 7.8 mm 6 months after ablative laser, and intralesional steroid injections. (B) Case 1: Dermoscopy showing a nodular mass with central hemorrhagic ulcer, serpentine-branched vessels on a pink structureless area, and remnants of brown pigmentation. (C) Case 3: Clinical image of a superficial spreading melanoma with a Breslow thickness of 0.75mm on the leg of a 33-year-old female. The lesion was first treated with chemical peels and reappeared after 3 months expanding continuously. (D) Case 3: Dermoscopic image showing a well demarcated lesion with chaos of border abruptness, eccentric gray-black structureless area, and white lines. (E) Case 4: Magnetic resonance imaging showing a melanoma metastasis in the brain causing severe edema and midline shift. The patient recalled non-surgical removal of a pigmented nevus from his leg

An 18-year-old female patient was referred to an experienced dermatologist because of an enlarged mass on the neck. The lesion had a history of 10 years. 2 years ago, ablative laser was performed for cosmetic reasons. The lesion enlarged in several months. This time the lesion was identified as a hypertrophic scar by the physician. He applied intralesional steroid therapy twice. The lesion enlarged continuously (Figure 1, A and B) and the patient sought professional medical advice. The lesion was excised, and the pathology confirmed nodular melanoma with a Breslow thickness of 7.8 mm, Clark level V. Sentinel lymph node biopsy showed extra capsular involvement

### Case 2

A 30-year-old male patient was referred to our department with the diagnosis of metastatic melanoma of unknown origin to examine the primary focus. The medical history revealed that a general surgeon removed a lesion on his neck for cosmetic reasons without sending the specimen to histopathological examination. The surgeon claimed the lesion as benign. After 2 years, the patient felt the enlargement of regional lymph nodes on the neck, and histopathology revealed a metastatic melanoma. Positron emission tomography showed metastasis in the lungs. After 1 year of treatment with BRAF and MEK inhibitors, the patient passed away.

### Case 3

A 33-year-old woman female requested therapeutic advice from a pharmacist for pigmented nevi on her left arm. The pharmacist recommended an acidic peeling to remove or destroy the pigmented area. After the application, the patient had a severe burn affecting the area, and the lesion was partially destroyed. However, after 3 months, the lesion started to evolve and expand (Figure 1, C and D). Histopathological analysis confirmed the diagnosis of superficial spreading melanoma with a Breslow thickness of 0.75mm and Clark level of III.

### Case 4

A 41-year-old male dentist had a history of increasing severity of headaches, vomiting and blurred vision for the last month. Cranial magnetic imaging revealed 3 masses in the brain. The most prominent lesion caused severe edema and midline shift (Figure 1 E). Positron emission tomography showed multiple masses, including masses at the level of the heart atrium, pancreas, lung, and subcutaneous tissue of the left leg. Histopathology of the mass from the subcutaneous mass in the leg was consistent with melanoma metastasis. The patient was referred to a dermatologist to examine the skin for the primary lesion. He mentioned a previous dermatology visit when he complained from a nevus on his leg 3 years ago. At the time, the physician removed the nevus with an ablative method. The patient is currently using BRAF+MEK inhibitors, and the large mass on the brain is treated with Gamma Knife radiosurgery.

## Discussion

The most common method used by non-dermatologist physicians to evaluate the malignancy potential is the ABCDE method (A-asymmetry, B-border, C-color, D-diameter, E-evolution). However, naked-eye assessment of a skin lesion using this method is inadequate. Clinical detection of a melanoma in early stages can be very challenging, even with dermoscopy. The examination of tumoral proliferations on the skin has improved significantly with the introduction of various dermoscopic algorithms [1–3]. Still, an initial melanoma can be evaluated as a nevus even by an experienced dermatoscopist and detected by digital dermoscopy follow-ups [4]. Besides, many clinically similar non-melanocytic lesions exist such as dermal nevi and nodular basal cell carcinomas [5]. A histopathological evaluation of the nevi is therefore always necessary before aesthetic treatment.

The risk of malpractice accusations in dermatology is comparatively low. However, malignant neoplasms of the skin require special considerations. Between 2006 and 2015 malignant neoplasms of the skin and melanoma together, ranked first in dermatology liability claims and resulted in the most extensive recovery in the US, probably reflecting the worldwide situation [6, 7].

The time lap between the destruction of the primary lesion and subsequent recurrence of a melanoma ranges between 2–10 years. This makes the accurate interpretation of the harm caused by this new wave, tricky [8]. The belief that one can easily differentiate a benign nevus from a malignant one possesses a significant risk with the frequent use of ablative modalities to treat skin lesions. We recommend professional organizations to determine a new policy that incorporates skin cancer education and use of dermoscopy in certifying treating physicians. Besides, a strict filing of the procedures and long-term follow-ups are needed to obtain the data allowing to measure the accuracy and safety of these techniques. Even with proper education and policies, a histopathologic evaluation of the lesion is crucial prior to conducting an aesthetic removal. We are extremely concerned by the widespread use of ablative techniques to remove skin lesions, as this may increase late melanomas. As physicians, our first rule when approaching a patient should always be as stated in the famous Latin phrase: “first, do no harm”.
